# 术后化疗胸腺肿瘤中的应用及对其预后的影响

**DOI:** 10.3779/j.issn.1009-3419.2016.07.10

**Published:** 2016-07-20

**Authors:** 可 马, 志涛 谷, 泳涛 韩, 剑华 傅, 毅 沈, 煜程 魏, 黎杰 谭, 鹏 张, 椿 陈, 仁泉 张, 印 李, 克能 陈, 和忠 陈, 永煜 刘, 有斌 崔, 允 王, 烈文 庞, 振涛 于, 鑫明 周, 阳春 柳, 媛 刘, 文涛 方, 中国胸腺肿瘤协作组成员

**Affiliations:** 1 610041 成都, 四川省肿瘤医院胸外科 Department of Thoracic Surgery, Sichuan Cancer Hospital, Chengdu 610041, China; 2 200030 上海, 上海交通大学附属上海胸科医院 Department of Thoracic Surgery, Shanghai Chest Hospital, Shanghai Jiao Tong University, Shanghai 200030, China; 3 510060 广州, 中山大学附属肿瘤医院胸外科 Department of Thoracic Surgery, Guangdong Esophageal Cancer Institute, Sun Yat-sen University Cancer Center, State Key Laboratory of Oncology in South China, Collaborative Innovation Center of Cancer Medicine, Guangzhou 510060, China; 4 266001 青岛大学医学院附属医院胸外科 Department of Thoracic Surgery, Affiliated Hospital of Qingdao University, Qingdao 266001, China; 5 200032 上海, 复旦大学附属中山医院胸外科 Department of Thoracic Surgery, Zhongshan Hospital, Fudan University, Shanghai 200032, China; 6 300052 天津, 天津医科大学附属总医院胸外科 Department of Endocrinology, Tianjin Medical University General Hospital, Tianjin 300052, China; 7 350001 福州, 福建医科大学附属协和医院胸外科 Department of Thoracic Surgery, Fujian Medical University Union Hospital, Fuzhou 350001, China; 8 230022 合肥, 安徽医科大学附属第一医院胸外科 Department of Thoracic Surgery, First Affiliated Hospital of Anhui Medical University, Hefei 230022, China; 9 450008 郑州, 郑州大学附属肿瘤医院胸外科 Department of Thoracic Surgery, Affiliated Cancer Hospital of Zhengzhou University, Zhengzhou 450008, China; 10 100142 北京, 北京大学附肿瘤医院胸外科 Department of Thoracic Surgery, Beijing Cancer Hospital, Beijing 100142, China; 11 200433 上海, 长海医院胸心外科 Department of Cardiothoracic Surgery, Changhai Hospital, Shanghai 200433, China; 12 110042 沈阳, 辽宁肿瘤医院胸外科 Department of Thoracic Surgery, Liaoning Cancer Hospital, Shenyang 110042, China; 13 130021 长春, 吉林大学附属第一医院胸外科 Department of Thoracic Surgery, First Affiliated Hospital of Jilin University, Changchun 130021, China; 14 610041 成都, 四川大学华西医院胸外科 Department of Thoracic Surgery, West China Hospital, Sichuan University, Chengdu 610041, China; 15 200032 上海, 复旦大学附属华山医院胸外科 Department of Thoracic Surgery, Huashan Hospital, Fudan University, Shanghai 200032, China; 16 300060 天津, 天津医科大学附属肿瘤医院食管癌中心 Department of Esophageal Cancer, Tianjin Cancer Hospital, Tianjin 300060, China; 17 310022 杭州, 浙江省肿瘤医院胸外科 Department of Thoracic Surgery, Zhejiang Cancer Hospital, Hangzhou 310022, China; 18 330006 南昌, 江西省人民医院胸外科 Department of Thoracic Surgery, Jiangxi People's Hospital, Nanchang 330006, China

**Keywords:** 胸腺肿瘤, 化疗, 手术, 预后, Thymic tumors, Chemotherapy, Surgery, Prognosis

## Abstract

**背景与目的:**

探讨术后化疗在胸腺肿瘤中的应用及术后化疗对Masaoka Ⅲ期/Ⅳ期预后的影响。

**方法:**

1994年3月至2012年12月, 中国胸腺瘤研究协作组(Chinese Alliance of Research for Thymomas, ChART)数据库共纳入2, 306例胸腺肿瘤病例, 资料相对完整1, 700例患者纳入本研究, 对其中Masaoka Ⅲ期/Ⅳ期665例患者进行进一步分析, 初步评估术后化疗的临床价值, 采用*Kaplan-Meier*法绘制不同亚组患者生存曲线, *Cox*回归进行多因素分析影响预后的因素。采用倾向值匹配研究(propensity-matched study, PSM), 评估化疗的临床价值。

**结果:**

1, 700例患者中未行术后化疗1, 406例(82.7%), 术后化疗294例(17.3%), 随着Masaoka分期的增加, 术后化疗患者的比例也随之增高, 差异有统计学意义(*P*<0.001)。对Masaoka Ⅲ期/Ⅳ期患者665例进行进一步分析, 其中未术后化疗组444例, 术后化疗组221例。两组患者在有无重症肌无力、WHO病理类型、病理分期、手术根治性、有无术后放疗等方面分布有差异(*P*<0.05)。其中C型胸腺瘤、不完全切除和术后放疗明显影响患者术后复发和生存(*P*<0.05)。术后化疗组5年和10年无病生存率分别为51%、30%, 5年和10年复发率分别为46%、68%, 而未术后化疗组5年和10年无病生存率分别为73%、58%。5年和10年复发率分别为26%、40%, 两组无病生存率和复发率均有明显统计学差异(*P*=0.001, *P*=0.001)。对有无重症肌无力, 病理类型, 病理分期, 手术根治性状态, 术后放疗等因素进行倾向值匹配筛选出其中158例未术后化疗和158例术后化疗共316例患者, 生存分析显示:未术后化疗组和术后化疗组两组5年生存率并无明显统计学差异(*P*=0.332)。

**结论:**

病理学类型、手术的根治性和术后放疗是影响进展期胸腺肿瘤患者术后生存和复发的主要因素。术后化疗并未给Masaoka-Koga Ⅲ期/Ⅳ期胸腺瘤患者带来生存获益。

## Abstract (en)

**Background and objective:**

To study the role of postoperative chemotherapy and its prognostic effect in Masaoka-Koga stage Ⅲ and Ⅳ thymic tumors.

**Methods:**

Between 1994 and 2012, 1, 700 patients with thymic tumors who underwent surgery without neoajuvant therapy were enrolled for the study. Among them, 665 patients in Masaoka-Koga stage Ⅲ and Ⅳ were further analyzed to evaluate the clinical value of postoperative chemotherapy. The *Kaplan-Meier* method was used to obtain the survival curve of the patients divided into different subgroups, and the *Cox* regression analysis was used to make multivariate analysis on the factors affecting prognosis. A Propensity-Matched Study was used to evaluate the clinical value of chemotherapy.

**Results:**

Two-hundred-twenty-one patients were treated with postoperative chemotherapy, while the rest 444 cases were not. The two groups showed significant differences (*P*<0.05) regarding the incidence of myasthenia gravis, World Health Organization (WHO) histological subtypes, pathological staging, resection status and the use of postoperative radiotherapy. WHO type C tumors, incomplete resection, and postoperative radiotherapy were significantly related to increased recurrence and worse survival (*P*<0.05). Five-year and 10-year disease free survivals (DFS) and recurrence rates in patients who underwent surgery followed by postoperative chemotherapy were 51% and 30%, 46% and 68%, comparing with 73% and 58%, 26% and 40% in patients who had no adjuvant chemotherapy after surgery (*P*=0.001, *P*=0.001, respectively). In propensity-matched study, 158 pairs of patients with or without postoperative chemotherapy (316 patients in total) were selected and compared accordingly. Similar 5-year survival rates were detected between the two groups (*P*=0.332).

**Conclusion:**

Pathologically higher grade histology, incomplete resection, and postoperative radiotherapy were found to be associated with worse outcomes in advanced stage thymic tumors. At present, there is no evidence to show that postoperative chemotherapy may help improve prognosis in patients with Masaoka-Koga-Koga stage Ⅲ and Ⅳ thymic tumors.

胸腺恶性肿瘤临床相对少见。其多发于前上纵隔，绝大多数患者经手术切除后可获得良好的疗效^[1]^，但约1/3的胸腺肿瘤患者就诊时即为进展期，肿瘤局部侵袭明显或发生远处转移无法经手术根治性切除，Masaoka等^[2]^报道局部进展期患者5年生存率约67%，而远处转移者为50%。胸腺肿瘤化疗仍有争议。化疗的目的一方面是降低肿瘤负荷为后续手术或放疗创造机会，另一方面则是延长疾病的控制时间。化疗可用于治疗的不同阶段，常采用的治疗模式包括术前化疗+手术、手术+术后化疗或放化疗。同时，对于发生远处转移的胸腺肿瘤患者，姑息性化疗常常是治疗的基本手段。目前胸腺肿瘤化疗没有标准治疗方案，有限的数据也多来源于不同单位采用各自的治疗模式和化疗方案的回顾性报道，因此结论千差万别。为此中国胸腺肿瘤协作组，收集国内18家医疗中心胸腺肿瘤数据，对近年来治疗胸腺肿瘤患者临床资（Chinese Alliance of Research for Thymomas, ChART）数据库中Masaoka Ⅲ期/Ⅳ期^[3]^胸腺肿瘤术后化疗病例的临床资料，初步探讨术后化疗在胸腺肿瘤治疗中的价值。

## 1 资料与方法

### 1.1 临床资料

1994年3月至2012年12月间ChART数据库共纳入2, 306例胸腺肿瘤病例, 删除分期不明确患者(224例)、WHO分型不明确患者(121例)、非手术患者(97例)、接受诱导放化疗患者(68例)、放疗有否不明确患者(96例), 共1, 700例患者纳入本研究。其中未行术后化疗1, 406例(82.7%), 术后化疗294例(17.3%)。取其中Masaoka Ⅲ期/Ⅳ期的病例, 以研究术后化疗对患者预后的影响。由于本研究中所有数据已去除个人信息, 伦理委员会放弃知情同意审查。

### 1.2 统计学方法

临床病例及随访资料由合作医疗中心录入数据库。采用SPSS 19.0软件进行统计学分析, 率的比较采用*χ*^2^检验。生存分析采用*Kaplan-Meier*法绘制生存曲线, *Log-rank*检验或者*Breslow*检验进行生存分析, *Cox*回归进行多因素分析影响预后的因素, 取95%可信区间。为减少本回顾性研究中不均衡因素的影响, 采用1:1卡钳法进行倾向值匹配研究, 比较术后化疗组和术后未化疗组间的生存率。*P*<0.05表示差异有统计学意义。

## 2 结果

### 2.1 术后化疗总体发生率

716例Ⅰ期患者中术后化疗39例(5.4%), 319例Ⅱ期患者中术后化疗34例(10.7%), 515例Ⅲ期患者中术后化疗137例(26.6%), 150例Ⅳ期患者中术后化疗84例(56%)(表 1)。本组资料中, 随着Masaoka分期的增加, 术后化疗患者的比例也随之增高, 有明显统计学差异(*P*<0.001), Ⅰ期/Ⅱ期患者总体化疗率10%左右(图 1、图 2)。

**1:** 不同分期术后化疗比例 Percentages of postoperative chemotherapy in different tumor stages Masaoka-Koga stage (n) Non-Chemo group (%) Chemo group (%) P value

Masaoka-Koga stage (*n*)	Non-Chemo group (%)	Chemo group (%)	*P* value
Ⅰ(716)	677(94.6)	39(5.4)	<0.001
Ⅱ(319)	285(89.3)	34(10.7)	
Ⅲ(515)	378(73.4)	137(26.6)	
Ⅳ(150)	66(44.0)	84(56.0)	
注:本表得到版权所有者©2011-2016 Journal of Thoracic Disease复制许可。			

**1:**
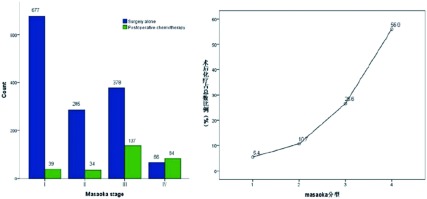
不同分期术后化疗比例 Percentage of postoperative chemotherapy in patients with different stage tumors

**2:**
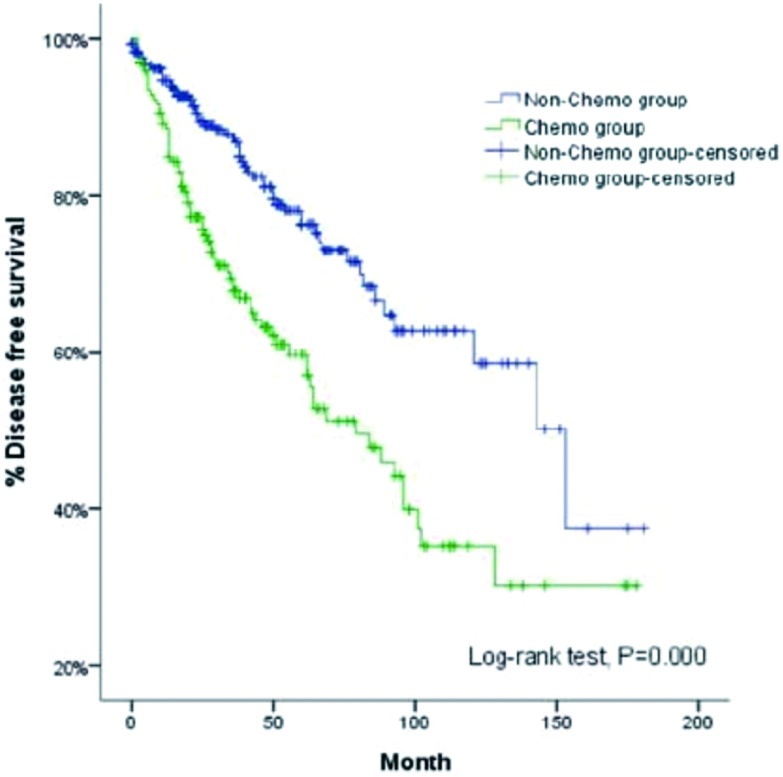
术后化疗组与未化疗组的总生存比较(*P*<0.001) Five-and ten-year disease free survivals (Non-Chemo group *vs* Chemo group, *P*<0.001)

### 2.2 Masaoka Ⅲ期/Ⅳ期患者的术后化疗

1, 700例中删除Ⅰ期(716例)和Ⅱ期(319例)患者, 对665例Masaoka Ⅲ期/Ⅳ期患者进行进一步分析, 其中未术后化疗组444例, 术后化疗组221例。

### 2.3 一般资料的临床分析

#### 2.3.1 患者临床特点及肌无力

结果显示: 未术后化疗组和术后化疗组在性别和年龄方面无明显差别, 但是术后未化疗组中肌无力患者明显较多(*P*<0.001)(表 2)。

**2:** 两组患者临床特点及肌无力分布情况Tab Clinical features and the distribution of myasthenia in the two groups.

Characteristics	Non-Chemo group, *n*=444 (%)	Chemo group, *n*=221 (%)	*P* value
Sex			0.493
Male	265 (65.8)	138 (34.2)	
Female	179 (68.3)	83 (31.7)	
Age, years	51.15	50.53	0.524
With or without myasthenia			<0.001
Yes	121 (87.7)	17 (12.3)	
No	323 (61.3)	204 (38.7)	
WHO types			<0.001
A	14 (87.5)	2 (12.5)	
AB	42 (87.5)	6 (12.5)	
B1	37 (86.0)	6 (14.0)	
B2	80 (87.0)	12 (13.0)	
B3	136 (71.6)	54 (28.4)	
C	123 (48.8)	129 (51.2)	
NETT	12 (50.0)	12 (50.0)	
WHO type (three classification)			<0.001
A+AB	56 (87.5)	8 (12.5)	
B1+B2+B3	253 (77.8)	72 (22.2)	
Tumor size (cm)	7.46	7.83	0.218
Pathological staging			<0.001
Ⅲ	378 (73.4)	137 (26.6)	
Ⅳ	66 (44.0)	84 (56.0)	
Resection status			<0.001
R0	326 (73.1)	120 (26.9)	
R1	47 (69.1)	21 (30.9)	
R2	71 (47.0)	80 (53.0)	
Other adjuvant therapies			<0.001
No	191 (43.0)	30 (13.9)	
Yes	253 (57.0)	186 (86.1)	
注：本表得到版权所有者©2011-2016 Journal of Thoracic Disease复制许可。			

#### 2.3.2 肿瘤类型

在不同WHO胸腺瘤病理分型亚组中采用术后化疗的比例有明显差异(*P*<0.001)。进一步分析显示C+NETT (neuroendocrine tumor)组术后化疗(51.1%)明显较B1+B2+B3组和A+AB组高(分别为22.2%和12.5%), 差异有统计学意义(*P*<0.001)(表 2)。

#### 2.3.3 肿瘤大小、病理分期、手术根治性之间的比较

肿瘤大小在术后化疗组和未化疗组无明显差异(*P*=0.218)。Ⅳ期患者术后化疗多于Ⅲ期患者(*P*<0.001)。本组资料总体手术根治性切除率为73.1%, 其中未化疗组明显高于化疗组, 差异有统计学意义(*P*<0.001)(表 2)。

#### 2.3.4 辅助治疗模式

本组数据中, 5例患者的放疗信息缺失, 对660例患者进行分析。191例患者单纯手术治疗(191/660, 28.9%), 其余患者接受术后辅助治疗(71.1%), 其中30例患者行术后单纯化疗(30/660, 4.5%), 253例行术后单纯放疗(253/660, 38.3%), 186例患者行术后放化疗(186/660, 28.2%)。未术后化疗组辅助放疗多于术后化疗组, 具有统计学差异(*P*<0.001)(表 2)。

### 2.4 Masaoka Ⅲ期/Ⅳ期患者生存相关因素分析

多因素分析显示:病理类型、手术根治性和术后辅助放疗是影响Ⅲ期/Ⅳ期胸腺肿瘤患者生存率的重要因素。C型胸腺瘤、不完全切除和联合术后放疗的患者生存预后更差(*P*=0.011, *P*=0.004, *P*=0.018)。未术后化疗组5年和10年无病生存率分别为73%、58%, 术后化疗组分别为生存率51%、30%, 两组结果有统计学差异(*P*<0.001)(表 3, 图 3)。

**3:** 生存相关因素的多因素分析 Multivariate analysis of survival-related risk factors

Risk factors	*P* value	OR(95%CI)
Myasthenia gravis(No *vs* Yes)	0.276	0.502(0.145-1.736)
Age(<50 yr *vs* ≥50 yr)	0.179	1.485(0.835-2.641)
Gender(Male *vs* Female)	0.737	0.902(0.495-1.645)
WHO histology type(A, AB/B1, B2 or B3/C)	0.011	
B1+B2+B3 *vs* A	0.577	1.541(0.337-7.041)
C *vs* A	0.067	3.952(0.908-17.195)
Masaoka-Koga stage(Ⅲ *vs* Ⅳ)	0.554	1.227(0.623-2.420)
Adjuvant chemotherapy(No *vs* Yes)	0.502	1.250(0.652-2.399)
Tumor size(≤5 cm *vs* > 5 cm)	0.876	1.056(0.531-2.100)
Complete resection(R0 *vs* R1+R2)	0.004	0.414(0.226-0.760)
Extent of thymectomy(Partial *vs* Total)	0.599	1.184(0.630-2.225)
Postoperative radiotherapy(No *vs* Yes)	0.018	0.451(0.233-0.873)
HR: hazard ratio; CI: confidence interval; WHO: World Health Organization. 注：本表得到版权所有者©2011-2016 Journal of Thoracic Disease复制许可。		

**3:**
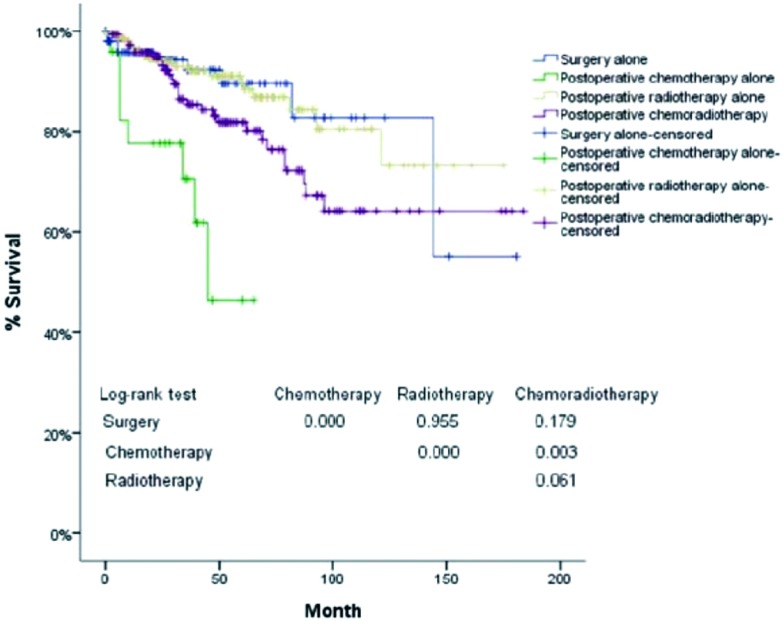
单纯手术、单纯化疗、术后放疗、术后放化疗亚组生存曲线 Survival curves for subgroups of patients with surgery alone, postoperative chemotherapy alone, postoperative radiotherapy alone, and postoperative chemoradiotherapy

进一步分层分析显示:Masaoka Ⅲ期/Ⅳ期胸腺肿瘤患者术后单纯化疗组生存率均明显差于单纯手术组、术后单纯放疗组合术后放化疗组(*P*<0.001, *P*<0.001, *P*=0.003)(表 4, 图 4)。

**4:** 各亚组生存率的比较 Comparison of survival rates among the different adjuvant therapy subgroups

*Log-rank*	Surgery alone			Postoperative chemotherapy alone			Postoperative radiotherapy alone			Postoperative chemoradiotherapy	
	*Chi-square*	Sig.		*Chi-square*	Sig.		*Chi-square*	Sig.		*Chi-square*	Sig.
Surgery alone				13.544	<0.001		0.003	0.955		1.805	0.179
Postoperative chemotherapy alone	13.544	<0.001					19.483	<0.001		8.604	0.003
Postoperative radiotherapy alone	0.003	0.955		19.483	<0.001					3.508	0.061
Postoperative hemoradiotherapy	1.805	0.179		8.604	0.003		3.508	0.061			
注：本表得到版权所有者©2011-2016 Journal of Thoracic Disease复制许可。											

**4:**
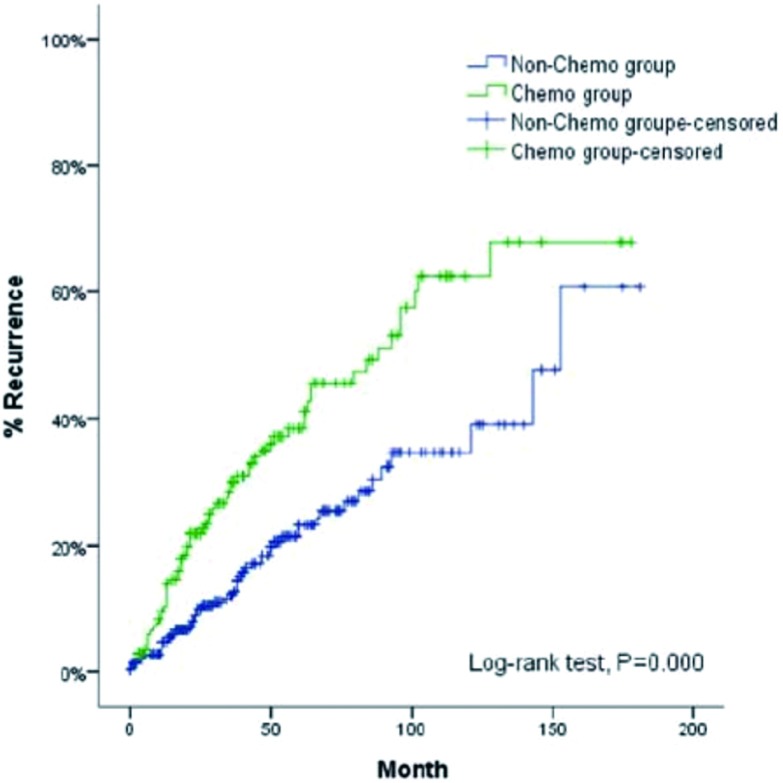
术后化疗组与未术后化疗组复发率的比较(*P*<0.001), 5年复查率分别为46%、26%, 10年复发率分别为68%、40%。 Five-and ten-year recurrence rates (Chemo group *vs* Non-Chemo group, *P*<0.001)

### 2.5 Masaoka Ⅲ期/Ⅳ期患者复发相关多因素分析

多因素分析显示:病理类型、手术根治性和术后放疗是影响Masaoka Ⅲ期/Ⅳ期胸腺肿瘤患者术后复发的重要因素, 其中C型胸腺瘤、不完全切除和联合术后放疗患者更容易出现复发(*P*=0.024, *P*=0.021, *P*=0.014)。未术后化疗组5年和10年复发率分别为26%、40%, 术后化疗组则分别46%、68%, 两组结果有统计学差异(*P*<0.001)(表 5, 图 5)。

**5:** MasaokaⅢ/Ⅳ患者复发相关多因素分析 Multivariate analysis of factors relating to recurrence in Masaoka-Koga stage Ⅲ AND Ⅳ patients

Factor	*P* value	OR
Myasthenia complication (No *vs* Yes)	0.090	0.466(0.193-1.127)
Age(<50 yr *vs* ≥50 yr)	0.344	1.218(0.809-1.833)
Sex(Male *vs* Female)	0.220	0.763(0.496-1.175)
WHO pathological type(A, AB/B1, B2 or B3/C)	0.024	
B1+B2+B3 vs A	0.277	1.809(0.621-5.268)
C *vs* A	0.037	3.083(1.069-8.887)
Masaoka-Koga stage(Ⅲ *vs* Ⅳ)	0.062	1.560(0.978-2.489)
Adjuvant chemotherapy(No *vs* Yes)	0.054	1.623(0.992-2.656)
Surgical approach(Thoracoscope *vs* Open)	0.641	1.411(0.332-5.993)
Tumor size(≤5 cm *vs* > 5 cm)	0.502	0.843(0.511-1.389)
Complete resection(R0 *vs* R1+R2)	0.021	0.617(0.410-0.929)
Postoperative radiotherapy(No *vs* Yes)	0.014	0.537(0.326-0.884)
注：本表得到版权所有者©2011-2016 Journal of Thoracic Disease复制许可。		

**5:**
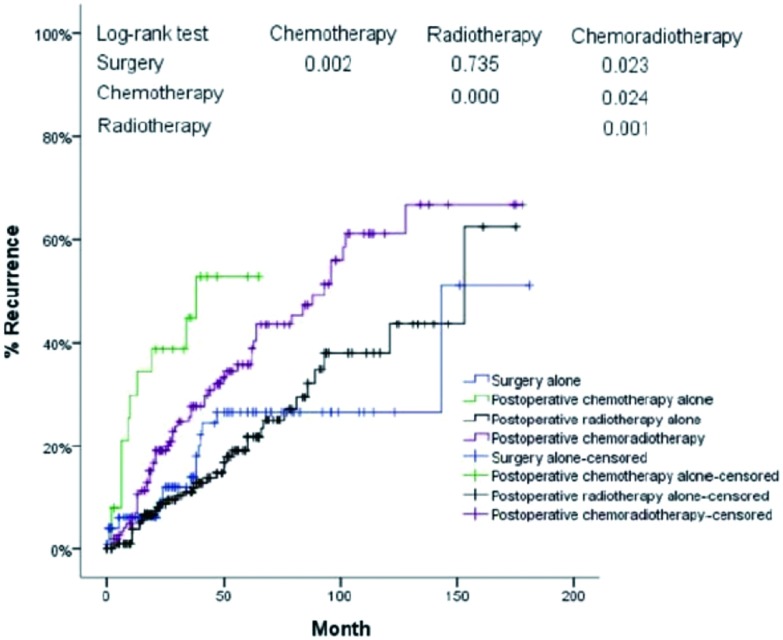
单纯手术、单纯化疗、术后放疗、术后放化疗亚组无疾病复发时间曲线 Cumulative incidence of recurrence for subgroups of patients with surgery alone, chemotherapy alone, radiotherapy alone, and chemoradiotherapy

进一步分层分析显示:Masaoka Ⅲ期/Ⅳ期胸腺肿瘤患者术后单纯化疗组复发率均明显高于单纯手术组、术后单纯放疗组合术后放化疗组(*P*=0.002, *P*<0.001, *P*=0.024)(表 6, 图 6)。

**6:** 各亚组复发率比较 Comparison of recurrence rates among different adjuvant therapy subgroups

*Log-rank*	Surgery alone			Postoperative chemotherapy alone			Postoperative radiotherapy alone			Postoperative chemoradiotherapy	
	*Chi-square*	Sig.		*Chi-square*	Sig.		*Chi-square*	Sig.		*Chi-square*	Sig.
Surgery alone				9.875	0.002		0.115	0.735		5.145	0.023
Postoperative chemotherapy alone	9.875	0.002					23.845	0.000		5.062	0.024
Postoperative radiotherapy alone	0.115	0.735		23.845	0.000					10.477	0.001
Postoperative chemoradiotherapy	5.145	0.023		5.062	0.024		10.477	0.001			
注：本表得到版权所有者©2011-2016 Journal of Thoracic Disease复制许可。											

**6:**
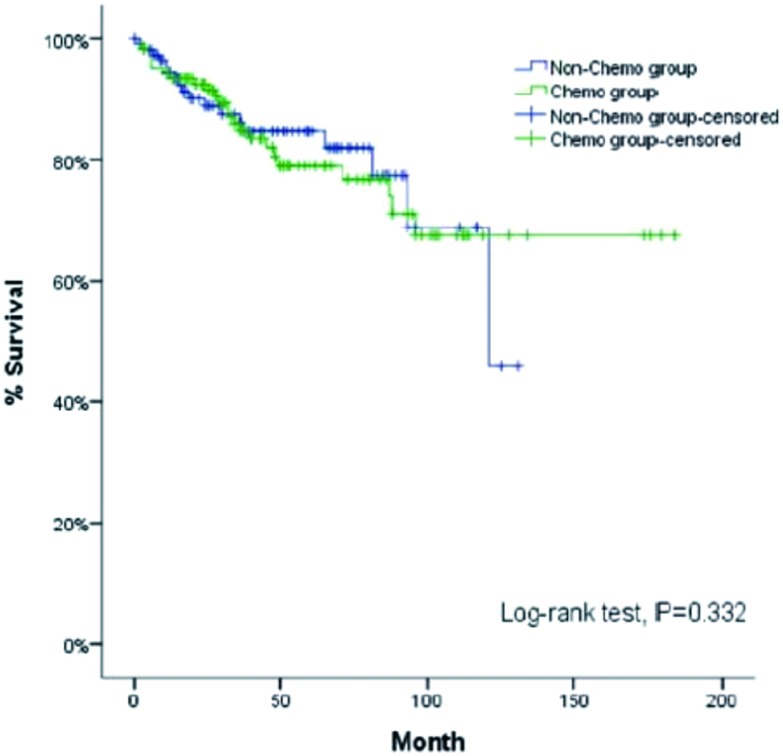
倾向性评分后两组的生存曲线 Survival curves of the two groups in the Propensity-Matched Study

### 2.6 术后化疗组和未化疗组之间的倾向值匹配研究

为减少未术后化疗组和术后化疗组之间不均衡因素对研究结果的影响。我们进一步采用1:1卡钳法倾向值匹配研究（Propensity-Matched Study, PSM），并行生存分析，匹配因素包括：有无重症肌无力，肿瘤分型，肿瘤病理分期，手术根治性状态，术后辅助放疗。获得158例未术后化疗和158例术后化疗共316例患者数据。其基本信息和生存曲线图如下（表 7）。

**7:** 1:1卡钳法倾向值匹配研究（Propensity-Matched Study, PSM） 1:1 Caliper Propensity-Matched Study results

Factor	Non-Chemo group (*n* =158)	Chemo group (*n* =158)	*P* value
Gender			0.816
Male	100	98	
Female	58	60	
Myasthenia gravis			0.489
Yes	21	17	
No	137	141	
WHO type three classifications			0.709
A+AB	8	8	
B1+B2+B3	62	55	
C+NETT	88	95	
Pathological staging			0.496
Ⅲ	121	126	
Ⅳ	37	32	
Resection status			0.224
R0	91	82	
R1	22	17	
R2	45	59	
Adjuvant radiotherapy			0.458
No	25	30	
Yes	133	128	
注：本表得到版权所有者©2011-2016 Journal of Thoracic Disease复制许可。			

结果显示: 经过倾向值匹配后, 未术后化疗组和术后化疗组总生存无统计学差异(*P*=0.332, 图 6)。因此, 术后化疗并未给Ⅲ期/Ⅳ期胸腺瘤患者带来生存获益。

## 3 讨论

胸腺肿瘤是少见的上皮源性肿瘤。其多发于前上纵隔，绝大多数患者经手术切除后可获得良好的疗效，但约1/3的胸腺肿瘤患者就诊时即为进展期，肿瘤局部侵袭明显或发生远处转移无法经手术根治性切除。

胸腺肿瘤患者的长期生存期差异很大。肿瘤病理分型、病理分期和手术的根治性是决定肿瘤患者生存的重要的因素。Kondo等^[4]^报道Ⅰ期、Ⅱ期、Ⅲ期、Ⅳ期胸腺患者术后不需要行辅助治疗^[5, 6]^。而Ⅲ期、Ⅳ期由于肿瘤局部侵袭或者肿瘤播散根治性切除率明显降低，常采用包括化疗在内的术后辅助治疗。本研究中显示：C型胸腺瘤、不完全切除和术后放疗是影响Masaoka Ⅲ/Ⅳ期患者生存和复发的重要因素。术后放疗患者中显示出更差的结果可能是由于该类患者中更多的是病理学侵袭性明瘤患者5年生存率分别为100%、98%、89%、71%。多数显，分期较晚的患者。不幸的是，在我们的试验中并未的研究结果也表明，对于完整切除的Ⅰ期、Ⅱ期胸腺肿瘤发现术后化疗给患者带来生存获益。

胸腺肿瘤术后化疗仍有争议。目前术后化疗更多是针对侵袭性胸腺肿瘤根治性术后预防性治疗, 或者非根治性手术后的辅助治疗, 多与放疗联合, 其目的一是降低肿瘤负荷, 增强疾病局部控制; 其二是延长复发和生存时间。目前胸腺肿瘤化疗没有标准治疗方案, 有限的数据也多来源于不同中心采用各自治疗方案的回顾性报道或小样本研究^[7-11]^。Ströbel等^[12]^回顾性分析228例胸腺瘤和胸腺鳞癌的治疗经验, 结果显示:术后化疗并不能改善A、AB、B1型胸腺瘤和Ⅱ期B2、B3型胸腺瘤的远期生存, 术后放疗可延长Ⅲ期胸腺瘤患者的生存时间。Kim等^[13]^对100例胸腺瘤临床资料分析发现术后放化疗与术后单独放疗相比, Ⅱ期和Ⅳ期胸腺瘤患者的5年生存率并无明显差异。Kondo等^[4]^对115个医疗中心治疗的1, 320例胸腺瘤分析后发现:术后放疗或术后化疗不能改善Ⅲ期/Ⅳ期根治性切除的胸腺瘤患者预后。同样, Attaran等^[14]^认为:尽管初始化疗和姑息化疗对某些患者显示出较好的治疗反应, 但目前仍并没有证据表明术后化疗能改善胸腺肿瘤患者的生存。我们使用ChART数据库进行回顾性分析后的结果与以往文献报道结果一致。本组资料也表明:对于所有Masaoka Ⅲ期/Ⅳ期的胸腺瘤及胸腺癌患者, 术后化疗并未在疾病复发和生存时间显示出优势。

由于本研究是采用回顾性数据, 各中心数据统计来源缺乏一致性。术后化疗组生存和复发相对更差的原因可能是由于该组患者中有较多高侵袭性病理学类型、Ⅳ期患者而导致不完全切除率增高。而上述因素均是导致胸腺肿瘤患者预后更差的原因。日本的一项来自115个分中心共186例患者的研究结果显示^[4]^:在完整切除的Ⅲ期/Ⅳ期胸腺瘤患者中术后化疗组、术后放化疗组、术后放疗组和单纯手术组的5年生存率分别为94.7%、80.9%、93.4%和100%。10年生存率分别为70.9%、70.4%、77.9%和95%, 单纯手术组与术后放化疗组5年生存率有明显差异(*P*=0.035, 3)。完整切除的Ⅲ期/Ⅳ期胸腺癌患者中单纯术后化疗组、术后放化疗组、术后放疗组和单纯手术组5年生存率分别为81.5%、46.6%、73.6%和72.2%, 术后放化疗组与术后放疗组、单纯手术组相比生存率均有明显差异(*P*=0.021, 3, *P*=0.039, 7)。在本组资料显示出:与单纯手术组和手术+放疗组相比, 术后放化疗组和术后化疗组, 生存更差。考虑到多中心回顾性研究数据的不均衡性, 我们尚不能得出化疗有害的结论, 但无论如何, 术后化疗并未给Masaoka Ⅲ期/Ⅳ期胸腺肿瘤患者带来任何生存益处。

为减少未术后化疗组和术后化疗组之间不均衡因素对研究结果的影响。我们进一步采用倾向值匹配研究, 并行生存分析, 匹配因素包括:有无重症肌无力, 肿瘤分型, 肿瘤病理分期, 手术根治性状态, 术后辅助放疗。同样的, 未术后化疗组和术后化疗组两组5年生存率并无明显统计学差异(*P*=0.332)。因此, 术后化疗并未给Ⅲ期/Ⅳ期胸腺瘤患者带来生存获益。

由于本研究是多中心参与, 时间跨度大, 所采用的化疗方案、化疗周期数、药物剂量各有不同, 因此无法评价各具体化疗方案的疗效, 只有前瞻性实验设计才能回答上述问题。我们的研究再次表明:肿瘤病理类型和手术根治性仍然是对包括进展期在内胸腺肿瘤患者预后起决定作用的因素。常规方案的术后化疗并未给局部晚期胸腺肿瘤患者带来生存获益。为改善患者预后, 更多的希望可能在于新辅助治疗和新药的研发方面。
